# Correction: Humoral immune response to inactivated COVID-19 vaccination at the 3rd month among people living with HIV

**DOI:** 10.1186/s12879-024-09913-w

**Published:** 2024-09-16

**Authors:** Songjie Wu, Shi Zou, Fangzhao Ming, Mengmeng Wu, Wei Guo, Zhongyuan Xing, Zhiyue Zhang, Jinli Liu, Weiming Tang, Weiming Tang

**Affiliations:** 1 Department of Nosocomial Infection Management, Zhongnan Hospital of Wuhan University, Hubei, China; 2 Wuhan Research Center for Infectious Diseases and Cancer, Chinese Academy of Medical Sciences, Wuhan, China; 3 Department of Infectious Diseases, Zhongnan Hospital of Wuhan University, Wuhan, Hubei 430071 China; 4 Wuchang District Center for Disease Control and Prevention, Wuhan, Hubei China; 5 Department of Pathology, Zhongnan Hospital of Wuhan University, Wuhan, China; 6 Department of Pathology, School of Basic Medical Sciences, Wuhan University, Wuhan, China; 7 School of Basic Medical Sciences, Wuhan University, Wuhan, China; 8 Guangdong Second Provincial General Hospital, Guangzhou, China; 9 The University of North Carolina at Chapel Hill Project-China, Guangzhou, 510095 China; 10 Hubei Engineering Center for Infectious Disease Prevention, Control and Treatment, Wuhan, China

Following publication of the original article [1], we have been notified of an error in affiliation number 8.

Originally published affiliation: Guangdong No. 2 Provincial People’s Hospital, Guangzhou, China.

Correct affiliation: Guangdong Second Provincial General Hospital, Guangzhou, China.
